# Daily cost-efficiency of robotic liver resection over laparoscopic and open liver resection especially for complex liver resection procedures: An inverse probability of treatment weighting analysis

**DOI:** 10.1007/s00595-026-03293-1

**Published:** 2026-04-22

**Authors:** Takeshi Kano, Yoshikuni Kawaguchi, Yujiro Nishioka, Akihiko Ichida, Takeshi Takamoto, Nobuhisa Akamatsu, Kiyoshi Hasegawa

**Affiliations:** Hepato-Biliary-Pancreatic Surgery Division, Department of Surgery, Graduate School of Medicine, The University of Tokyo, 7-3-1 Hongo, Bunkyo-ku, Tokyo, 113-8655 Japan

**Keywords:** Health Care Costs, Hepatectomy, Hospital Costs, Minimally Invasive Surgical Procedures, Robotic Surgical Procedures

## Abstract

**Purpose:**

To address our hypothesis that increased surgical costs for robotic liver resection would be compensated by reduced postoperative care expenses, we compared the economic outcomes of robotic, laparoscopic, and open liver resection under the Japanese national health insurance system.

**Methods:**

Patients who underwent liver resection between 2022 and 2024 were included in this study. The perioperative and economic outcomes were compared among the patients who underwent robotic, laparoscopic, and open liver resection using inverse probability of treatment weighting. Subgroup analyses were conducted for single partial resection, mono-segmentectomy, and the resection of two contiguous sections.

**Results:**

Of 402 patients, 302 met the inclusion criteria: robotic (*n* = 45), laparoscopic (*n* = 75), and open (*n* = 182). The hospital revenue per admission did not differ significantly among the three groups (*P* = 0.427). The overall hospital profit per day was significantly higher in the robotic group than in the laparoscopic and open groups (*P* < 0.001). The hospital profit per day was more favorable in patients undergoing robotic liver resection for mono-segmentectomy than in those undergoing laparoscopic and open liver resection, and for the resection of two contiguous sections than for open liver resection.

**Conclusion:**

Although the hospital revenue and profit per admission did not differ significantly among the groups, robotic liver resection may offer economic advantages regarding hospital profit per day.

**Supplementary Information:**

The online version contains supplementary material available at 10.1007/s00595-026-03293-1.

## Introduction

Robotic liver resection (RLR) is increasingly performed worldwide. Studies have reported that RLR is associated with fewer postoperative complications and improvements in postoperative recovery compared to open liver resection (OLR) [1, 2] and a lower open conversion rate [3–5], less blood loss [5], and shorter hospital stay [3, 5].

However, RLR may be associated with increased surgical costs and hinder hospital financial performance because the introduction of RLR requires high initial capital investment and maintenance costs for the robotic platform. Previous studies have investigated the economic outcomes of RLR [1, 6–20]. All these studies were conducted in the United States, Europe, Canada, and other Asian countries, excluding Japan. No study has evaluated the economic outcomes of RLR under the Japanese national health insurance system. Because healthcare infrastructures and resource allocation differ across international settings, direct cost comparisons are complicated. We hypothesized that higher total RLR costs would be compensated by reduced costs for postoperative care due to better postoperative outcomes with RLR than with laparoscopic liver resection (LLR) or OLR.

To address our hypothesis, we compared the economic outcomes of RLR, LLR, and OLR under the Japanese national health insurance system, focusing on the critical indicators for hospital management, including hospital profit and hospital operating margin per admission.

## Methods

### Study population

A prospectively maintained database was reviewed to identify patients who underwent liver resection at The University of Tokyo Hospital between January 2022 and December 2024. The patients who underwent concomitant procedures other than liver resection were excluded to evaluate the economic outcomes of liver resection alone. We also excluded patients who underwent liver resection with extrahepatic bile duct resection because the Japanese national health insurance system, as of December 2024, did not cover the procedure for laparoscopic or robotic liver resection. This study was approved by the Research Ethics Committee/Institutional Review Board of The University of Tokyo (approval number: 2158-11) and followed the principles outlined in the Declaration of Helsinki. Informed consent was obtained using an opt-out approach on our institutional website.

## Definition

Liver resections were classified into three levels of difficulty based on the three-level complexity classification, which considers the tumor location and tumor size [21–24]: Grade I, wedge resection for anterolateral or posterosuperior segment and left lateral sectionectomy; Grade II, segmentectomy of the anterolateral segment and left hepatectomy; and Grade III, posterosuperior segmentectomy, right posterior sectionectomy, right hepatectomy, central hepatectomy, and extended left/right hepatectomy. Postoperative complications, including both the inpatient and outpatient periods, were assessed within 90 days after surgery and graded according to the Clavien-Dindo (CD) classification [25]. The postoperative hospital stay was measured from the day of surgery to the day of discharge.

## Indications and surgical procedures for RLR, LLR, and OLR

At The University of Tokyo Hospital, OLR has been the standard approach. We started LLR in 2008 and RLR in 2021 with the increasing prevalence of minimally invasive liver resection (MILR) and the introduction of national insurance system coverage for MILR. We prioritized the safe introduction of MILR and initiated Grade I liver resection, as defined by the three-level complexity classification. The indications for MILR gradually expanded to include technically complex Grade II and III procedures as the learning curve was overcome [26].

The details of the surgical procedures for RLR, LLR, and OLR have been described elsewhere [27, 28]. In short, the extent of liver resection was determined based on a comprehensive preoperative liver function assessment, including serum bilirubin levels, presence of uncontrolled ascites, and ICG retention rate at 15 min, regardless of underlying cirrhosis or portal hypertension. All surgical procedures were performed under a combination of general and epidural anesthesia. We performed OLR through either a J-shaped or inverted L-shaped laparotomy incision, LLR through a 4-cm umbilicus incision with a multiple access device and three ports, and RLR through a 5-cm umbilicus incision with a multiple access device and two ports [27]. For RLR, the da Vinci^®^ Surgical System (Intuitive Surgical, Inc., Sunnyvale, CA, USA) was primarily used, and the hinotori™ Robotic System (Medicaroid Corporation, Kobe, Japan) was introduced in 2024. We employed the clamp crushing method for parenchymal transection using Pean forceps and a bipolar vessel-sealing device (LigaSure; Medtronic, Boulder, CO, USA) for OLR, an ultrasonic coagulation and cutting device (THUNDERBEAT; Olympus Corporation, Tokyo, Japan) for LLR, and the double-hand crush method with double Maryland bipolar forceps for RLR [27]. A cavitron ultrasonic surgical aspirator (CUSA) (Integra LifeSciences, Plainsboro, NJ, USA) was not used for all types of liver resections. Intermittent inflow occlusion was applied if necessary. Based on the individual case requirements, surgical drain placement was determined at the surgeon’s discretion. The number of assistant surgeons was generally two or three for OLR, two for LLR, and one for RLR.

## Postoperative management

Standardized postoperative management for patients undergoing liver resection has been described elsewhere [23]. Additionally, we implemented our Enhanced Recovery After Surgery program, named the Fast-Track Return to Normal Activity (FASTOL) program, in patients undergoing minimally invasive surgery in 2022 [27]. On postoperative day (POD) 1, oral intake and mobilization exercises were initiated. Epidural anesthesia was administered for no more than POD 5 and/or intravenous and oral analgesia were used. Steroids, proton pump inhibitors, and diuretics were routinely administered. Prophylactic antibiotics were administered for no longer than 48 h after the operation. Blood tests and chest/abdominal radiographs were routinely performed on PODs 1 and 3. The drainage tubes were removed on POD 1, unless the patients had bile leaks or infectious fluid collection. The criteria for hospital discharge were as follows: no postoperative complications were observed, intravenous analgesia was not needed, and the patients could perform the activities of daily living. The first visit to the outpatient clinic was scheduled approximately 14 days after hospital discharge.

## Calculation of hospital revenue and costs in Japan

The hospital revenue per admission was calculated using the Japanese lump-sum payment system according to the Diagnostic Related Groups/Prospective Payment System, which is the Japanese equivalent of the system in the United States [29, 30]. Briefly, the payment system comprised a Diagnosis Procedure Combination (DPC) component, which includes the hospital basic fee, charges for medications and examinations, and a fee-for-service component, which includes charges for surgical procedures and anesthesia. The Japanese national health insurance system sets the surgical fee for liver resection procedures of OLR, LLR, and RLR on the basis of the anatomical extent of liver resection (Table S1 [Online Resource 1]). The Japanese national health insurance system does not cover LLR and RLR with extrahepatic bile duct resection and reconstruction. The daily hospital basic fee within the lump-sum payment decreases gradually on the three levels depending on the duration of hospital admission [29]. In general, 70–90% of the hospital revenue is covered by the Japanese national health insurance system, with the percentage mainly depending on the patient age.

The hospital costs per admission were calculated as the sum of the following costs: devices, instruments, robotic arms, and medications, which were not reimbursed in the DPC lump-sum payment. The hospital profit was calculated as hospital revenue minus hospital costs for treatment, and the hospital profit per day was obtained by dividing the hospital profit by the length of the postoperative hospital stay. The hospital operating margin was calculated by dividing the hospital profit by hospital revenue per admission.

The intraoperative surgeon labor costs were estimated as the operation time multiplied by the hypothetical hourly wage of doctors in national university hospitals in Japan according to the Hospital Management Accounting System 2 [31] because the labor expenses specific to surgeons were not claimed in the Japanese healthcare system. The initial capital investments of the robotic system were incorporated into the analysis as depreciation costs per surgery on a per-case basis using a straight-line depreciation method, calculated by the robot system price, maintenance cost, and the expected working duration. The economic analysis, including the above two estimated expenses, is shown as a supplemental evaluation in the Online Resources because the expenses for surgeons and robotic depreciation were not clearly defined and because the depreciation costs of the laparoscopic camera system were not estimated.

Given that hospital revenue per admission was influenced by a fee-for-service component (i.e., types of liver resection procedures), economic outcomes were further assessed in patients undergoing single partial liver resection, mono-segmentectomy, and resection of two contiguous sections without vascular reconstruction.

Economic outcomes calculated in Japanese yen were converted into U.S. dollars at an exchange rate of 140 yen per dollar.

### Statistical analysis

Continuous variables related to the economic outcomes are expressed as the mean ± standard deviation (SD) and were compared between groups using the One-way Analysis of Variance and the Jonckheere–Terpstra test. Continuous variables not related to economic outcomes are expressed as medians (interquartile range [IQR]) and were compared between groups using the Kruskal-Wallis test. Categorical variables are expressed as numbers (%) and compared using Fisher’s exact test.

To perform inverse-probability of treatment weighted (IPTW) analyses, the propensity score for each patient was calculated as the predicted probability of RLR, LLR, and OLR from a multinomial logistic regression model [32, 33]. The multinomial regression model included the following demographics and clinical characteristics: age, sex, body mass index (BMI; > 25 kg/m^2^), American Society of Anesthesiologists physical status score (≥ 3), three-level complexity classification, liver resection procedure category used in the Japanese national health insurance system, the largest diameter of tumors, disease type, and number of resection sites. A sandwich variance estimator was used to estimate the standard error in the IPTW analysis to account for the weighting [34–36]. To assess monotonic trends across ordinal treatment groups while accounting for sampling weights, a rank-based trend test was used. To assess covariate balance across the three groups, standardized mean differences (SMDs) were calculated for each pair of groups (pairwise comparisons) before and after IPTW.

All statistical tests were two-sided, and statistical significance was set at *P* < 0.05. Statistical analyses were performed using SAS version 9.4 (SAS Institute Inc., Cary, NC, USA) and R version 4.2.1 (R Foundation for Statistical Computing, Vienna, Austria).

## Results

### Demographics and clinical characteristics by the liver resection approach

Of the 402 patients who underwent liver resection, 302 met the inclusion criteria (the RLR group, *n* = 45; the LLR group, *n* = 75; and the OLR group, *n* = 182) (Fig. S1 [Online Resource 2]). The proportion of RLR vs. LLR vs. OLR was 6% vs. 23% vs. 71% in 2022, 16% vs. 37% vs. 47% in 2023, and 27% vs. 32% vs. 41% in 2024. Table 1 presents the patients’ demographics and clinical characteristics. Sex and body mass index (BMI) differed significantly among the groups (*P* = 0.030 and *P* = 0.009, respectively). The liver resection complexity grades differed significantly different among the groups: Grade II (intermediate complexity) /III (high complexity) procedures were common (63.2%) in the OLR group, intermediate (48.8%) in the RLR group, and low (37.4%) in the LLR group. According to the liver resection procedures stratified by the surgical charge system in Japan, single partial resection and mono-segmentectomy were frequently performed across the groups. After IPTW adjustment, the intergroup differences were balanced (Table 2).

**Table 1:** Demographics and clinical characteristics by the liver resection approach before inverse-probability of treatment weighted adjustment

Characteristics	RLR group *N* = 45	LLR group *N* = 75	OLR group *N* = 182	SMD	*P* value
Age, median (IQR), year	71 (60–76)	71 (59–76)	66.5 (57–75)	0.205	0.130
Sex, Male/Female, n	35/10	47/28	142/40	0.335	0.038
BMI, median (IQR), kg/m^2^	25.2 (23.5–26.4)	23.4 (20.8–25.9)	23.0 (20.4–25.5)	0.193	0.009
BMI > 25 kg/m^2^, n (%)	24 (53.3)	24 (32.0)	54 (29.7)	0.441	0.013
ASA physical status score ≥ 3, n (%)	5 (11.1)	19 (25.3)	34 (18.7)	0.213	0.15
Child–Pugh classification, n (%)				0.261	0.501
A	45 (100.0)	72 (96.0)	176 (96.7)		
B	0 (0.0)	3 (4.0)	6 (3.3)		
ICG–R15, median (IQR), %	9.9 (6.4–12.9)	8.7 (5.6–13.6)	9.4 (5.6–13.7)	0.114	0.898
Three–level complexity classification^a^, n (%)				0.291	< 0.001
Grade I	23 (51.1)	47 (62.7)	67 (36.8)		
Grade II	11 (24.4)	14 (18.7)	22 (12.1)		
Grade III	11 (24.4)	14 (18.7)	93 (51.1)		
Liver resection procedures stratified by the surgical charge system in Japan, n (%)				0.351	< 0.001
Partial liver resection, single resection	13 (28.9)	51 (68.0)	54 (29.7)		
Partial liver resection, multiple resection	7 (15.6)	6 (8.0)	51 (28.0)		
Mono–segmentectomy	15 (33.3)	7 (9.3)	30 (16.5)		
Left lateral sectionectomy	2 (4.4)	8 (10.7)	2 (1.1)		
Resection of one section^b^	2 (4.4)	1 (1.3)	20 (11.0)		
Resection of two contiguous sections without vascular reconstruction	6 (13.3)	2 (2.7)	25 (13.7)		
Disease type, n (%)				0.183	0.329
HCC	26 (57.8)	30 (40.0)	70 (38.5)		
ICC	2 (4.4)	7 (9.3)	11 (6.0)		
CRLM	11 (24.4)	28 (37.3)	73 (40.1)		
NET	3 (6.7)	1 (1.3)	6 (3.3)		
Others	3 (6.7)	9 (12.0)	22 (12.0)		
Largest tumor diameter, median (IQR), cm	2.2 (1.5–3.2)	2.5 (1.7–4.0)	3.0 (1.6–5.1)	0.331	0.064
Number of resection sites > 2, n (%)	5 (11.1)	3 (4.0)	65 (35.7)	0.607	< 0.001

Abbreviations: RLR, robotic liver resection; LLR, laparoscopic liver resection; OLR, open liver resection; SMD, standardized mean difference; IQR, interquartile range; BMI, body mass index; ASA, American Society of Anesthesiologists; ICG–R15, indocyanine green retention rate at 15 min, HCC, hepatocellular carcinoma; CRLM, colorectal liver metastases; ICC, intrahepatic cholangiocarcinoma; NET, neuroendocrine tumor

^a^ Kawaguchi Y, et al. Ann Surg, 2018; Clin Liver Dis. 2020

^b^Excluding left lateral segmentectomy

**Table 2:** Demographics and clinical characteristics by the liver resection approach after inverse-probability of treatment weighted adjustment

Characteristics	RLR group	LLR group	OLR group	SMD	*P* value
Age > 80 years, %	9.0	13.0	11.7	0.090	0.888
Sex, Male/Female, %	86.0/14.0	79.0/21.0	74.6/25.4	0.184	0.439
BMI > 25 kg/m^2^, %	61.7	43.7	32.3	0.360	0.183
ASA physical status score ≥ 3, %	24.3	12.5	16.5	0.194	0.520
Child–Pugh classification, %				0.238	0.538
A	100.0	97.0	97.2		
B	0.0	3.0	2.8		
ICG–R15 > 10%, %	43.9	36.1	42.7	0.133	0.858
Three–level complexity classification^a^, %				0.074	0.823
Grade I	50.1	40.8	41.9		
Grade II	7.4	14.2	16.8		
Grade III	42.5	45.0	41.3		
Liver resection procedures stratified by the surgical charge system in Japan, %				0.116	0.570
Partial liver resection, single resection	38.6	41.1	40.1		
Partial liver resection, multiple resection	37.7	37.7	21.8		
Mono–segmentectomy	12.6	10.7	7.6		
Left lateral sectionectomy	2.7	4.1	17.7		
Resection of one section^b^	1.6	0.7	7.6		
Resection of two contiguous sections without vascular reconstruction	6.8	5.7	10.3		
Disease type, %				0.183	0.884
HCC	33.6	52.0	42.3		
ICC	4.1	8.9	7.5		
CRLM	46.1	28.5	37.1		
NET	1.7	0.8	2.8		
Others	0.0	9.8	10.2		
Largest tumor diameter, median (IQR), cm	3.4 (2.0–6.0)	3.6 (2.0–4.5)	2.5 (1.5–5.0)	0.079	0.657
Number of resection sites > 2, %	38.9	32.5	25.3	0.159	0.732

Abbreviations: RLR, robotic liver resection; LLR, laparoscopic liver resection; OLR, open liver resection; SMD, standard mean difference; IQR, interquartile range; BMI, body mass index; ASA, American Society of Anesthesiologists; ICG–R15, indocyanine green retention rate at 15 min, HCC, hepatocellular carcinoma; CRLM, colorectal liver metastases; ICC, intrahepatic cholangiocarcinoma; NET, neuroendocrine tumor

^a^ Kawaguchi Y, et al. Ann Surg, 2018; Clin Liver Dis. 2020

^b^ Excluding left lateral segmentectomy

### Surgical and postoperative outcomes by the liver resection approach with and without IPTW adjustment

Table 3 shows surgical and postoperative outcomes with and without IPTW adjustment. The operative time and the estimated blood loss (EBL) were significantly different among the groups. Postoperative complications ≥ CD classification grade III, including both inpatient and outpatient periods were significantly different among the groups: 0.0%, 7.3%, and 13.1% in the RLR, the LLR, and the OLR groups, respectively (*P* < 0.001). The median (IQR) length of postoperative hospital stay was significantly different among the groups: 4 (4–5) days in the RLR group, 6 (4–7.5) days in the LLR group, and 9 (7–13) days in the OLR group (*P* < 0.001). The 30-day unplanned readmission rate was not significantly different among the groups. After IPTW adjustment, operative time, EBL and postoperative hospital stay remained statistically significant among the groups.

**Table 3:** Surgical and postoperative outcomes by the liver resection approach with and without inverse-probability of treatment weighted adjustment

Characteristics	RLR group *N* = 45	LLR group *N* = 75	OLR group *N* = 182	*P* value
Without adjustment				
Operative time, median (IQR), min	388 (313–466)	272 (180–319)	334(253–417)	< 0.001
Estimated blood loss, median (IQR), ml	50 (0–235)	163 (50–408)	560 (310–1014)	< 0.001
Morbidity Clavien–Dindo classification^a^, n (%)				< 0.001
No morbidity	39 (86.7)	62 (82.7)	108 (59.3)	
Grade I or II	6 (13.3)	11 (14.7)	50 (27.5)	
Grade III or IV	0 (0.0)	2 (2.7)	24 (13.2)	
Postoperative hospital stay, median (IQR), day	4 (4–5)	6 (4–7.5)	9 (7–13)	< 0.001
30-day readmission, n (%)	1 (2.2)	0 (0.0%)	5 (2.7)	0.413
With adjustment				
Operative time, median (IQR), min	393 (321–527)	319 (252–457)	316(247–411)	< 0.001
Estimated blood loss, median (IQR), ml	100 (30–120)	410 (120–1320)	460 (270–970)	< 0.001
Morbidity Clavien–Dindo classification^a^, %				0.708
No morbidity	63.0	62.1	61.1	
Grade I or II	37.0	30.6	25.8	
Grade III or IV	0.0	7.3	13.1	
Postoperative hospital stay, median (IQR), day	5 (4–8)	5 (5–7)	8 (7–13)	< 0.001
30-day readmission, %	3.1	0.0	3.2	0.528

Abbreviations: RLR, robotic liver resection; LLR, laparoscopic liver resection; OLR, open liver resection; IQR, interquartile range

^a^ Clavien PA, Barkun J, de Oliveira ML, et al. Ann Surg. 2009; 250:187–196

### Overall economic outcomes by the surgical approach after IPTW adjustment

**Fig. 1:**
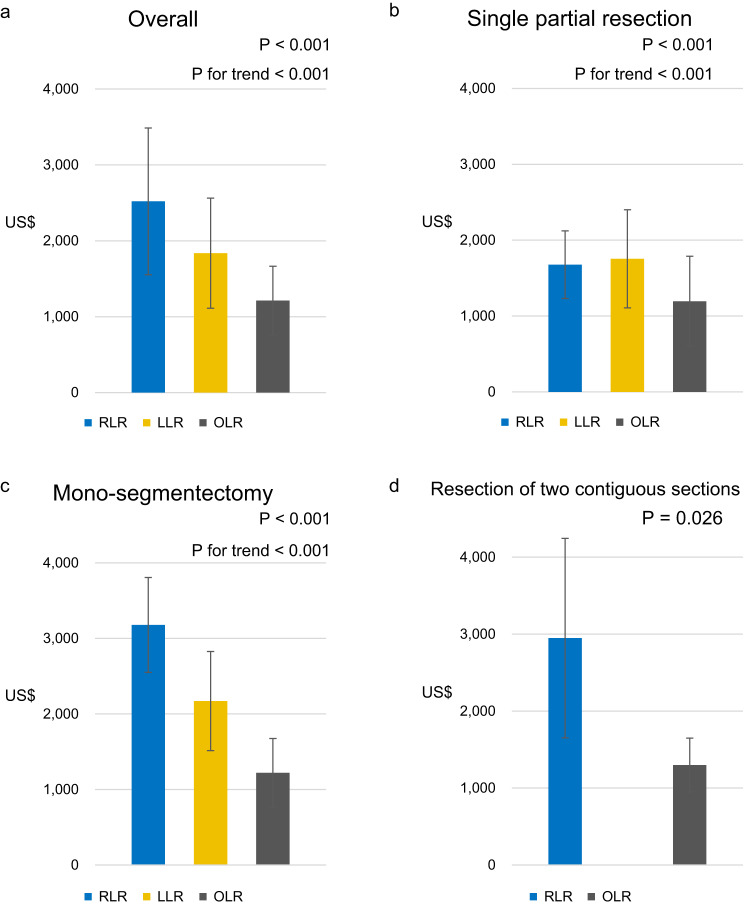
Hospital profit per day after inverse-probability of treatment weighted adjustment **(a)** RLR vs. LLR vs. OLR; Overall cohort, 2,520 ± 967 US$ vs. 1,836 ± 726 US$ vs. 1,231 ± 451 US$ (*P* < 0.001 and P for trend < 0.001) **(b)** RLR vs. LLR vs. OLR; Single partial resection, 1,677 ± 447 US$ vs. 1,755 ± 646 US$ vs. 1,195 ± 593 US$ (*P* < 0.001 and P for trend < 0.001) **(c)** RLR vs. LLR vs. OLR; Mono-segmentectomy, 3,178 ± 628 US$ vs. 2,171 ± 656 US$ vs. 1,221 ± 453 US$ (*P* < 0.001 and P for trend < 0.001) **(d)** RLR vs. OLR; resection of two contiguous sections, 2,948 ± 1,296 US$ vs. 1,299 ± 349 US$ (*P* = 0.026) Abbreviations: RLR, robot-assisted liver resection; LLR, laparoscopic liver resection; OLR, open liver resection

Table 4 shows overall economic outcomes by the surgical approach. The mean ± SD total hospital revenue per admission were not significantly different among the RLR, LLR, and OLR groups: 13,434 ± 2,797 US$ vs. 12,507 ± 3,843 US$ vs. 13,161 ± 4,628 US$ (*P* = 0.427). The mean ± SD hospital costs per admission were significantly different among the groups: 2,919 ± 1,589 US$ vs. 1,958 ± 1,088 US$ vs. 1,661 ± 1,111 US$ (*P* < 0.001). The mean ± SD hospital profit was not significantly different among the groups: 10,514 ± 3,163 US$ vs. 10,543 ± 3,502 US$ vs. 11,500 ± 4,006 US$ (*P* = 0.100). The median hospital operating margin was higher than 0.8 in all the groups. The mean ± SD hospital profit per day was significantly different among the groups and significantly decreased from the RLR to LLR to OLR groups: 2,520 ± 967 US$ vs. 1,836 ± 726 US$ vs. 1,213 ± 451 US$ (*P* < 0.001 and P for trend < 0.001) (Fig. 1a).

**Table 4:** Overall economic outcomes by the liver resection approach after inverse-probability of treatment weighted adjustment

Characteristics	RLR group	LLR group	OLR group	*P* value
Hospital revenue per admission, mean ± SD				0.427
Yen	1,880,711 ± 391,510	1,751,007 ± 538,017	1,842,559 ± 647,883	
US$^a^	13,434 ± 2,797	12,507 ± 3,843	13,161 ± 4,628	
Hospital costs per admission, mean ± SD				< 0.001
Yen	408,682 ± 222,430	274,151 ± 152,259	232,489 ± 155,498	
US$^a^	2,919 ± 1,589	1,958 ± 1,088	1,661 ± 1,111	
Hospital profit per admission, mean ± SD				0.100
Yen	1,472,029 ± 442,883	1,476,856 ± 490,244	1,610,070 ± 560,844	
US$^a^	10,514 ± 3,163	10,549 ± 3,502	11,500 ± 4,006	
Hospital operating margin, median (IQR)	0.80 (0.73–0.81)	0.84 (0.84–0.90)	0.89 (0.85–0.90)	< 0.001

Abbreviations: RLR, robotic liver resection; LLR, laparoscopic liver resection; OLR, open liver resection; SD, standard deviation; IQR, interquartile range

^a^ Calculated on an exchange rate of 140 yen to the dollar (the average from 2022 to 2024)

Table S2 (Online Resource 3) presents economic evaluations, including the estimated intraoperative surgeon labor costs and estimated depreciation costs per surgery, for the da Vinci^®^ Surgical System (1,609 US$) and the hinotori™ Robotic System (1,265 US$). The median hospital operating margin was 0.61 in the RLR group but > 0.8 in the other groups. The mean ± SD intraoperative surgeon labor costs were significantly different among the groups: 501 ± 158 US$ vs. 427 ± 196 US$ vs. 648 ± 292 US$ (*P* < 0.001). The mean ± SD hospital profit per day was significantly different among the groups and significantly decreased from the RLR to LLR to OLR groups: 2,013 ± 890 US$ vs. 1,760 ± 711 US$ vs. 1,142 ± 436 US$ (*P* < 0.001 and P for trend < 0.001) (Fig. S2a [Online Resource 4]).

### Economic outcomes by the liver resection procedure types after IPTW adjustment

Table 5 shows the economic outcomes in patients undergoing single partial liver resection, mono-segmentectomy, and the resection of two contiguous sections (LLR was not assessed in the last subgroup because only 2 patients underwent this category of the LLR procedure). The mean ± SD hospital profit per day was significantly different among the groups and significantly decreased from the RLR to LLR to OLR groups: RLR vs. LLR vs. OLR; single partial resection, 1,677 ± 447 US$ vs. 1,755 ± 646 US$ vs. 1,195 ± 593 US$ (*P* < 0.001 and P for trend < 0.001) (Fig. 1b); mono-segmentectomy, 3,178 ± 628 US$ vs. 2,171 ± 656 US$ vs. 1,221 ± 453 US$ (*P* < 0.001 and P for trend < 0.001) (Fig. 1c); RLR vs. OLR; resection of two contiguous sections, 2,948 ± 1,296 US$ vs. 1,299 ± 349 US$ (*P* = 0.026) (Fig. 1d).

**Table 5:** Economic outcomes by the liver resection procedure types after inverse-probability of treatment weighted adjustment

Characteristics	RLR group	LLR group	OLR group	*P* value
	** Single partial resection**			
Hospital revenue per admission, mean ± SD				0.703
Yen	1,541,261 ± 259,082	1,578,583 ± 324,866	1,673,255 ± 917,653	
US$^a^	11,009 ± 1,851	11,276 ± 2,320	11,952 ± 6,555	
Hospital costs per admission, mean ± SD				< 0.001
Yen	408,496 ± 144,714	255,520 ± 153,237	224,733 ± 151,982	
US$^a^	2,918 ± 1,034	1,825 ± 1,095	1,605 ± 1,086	
Hospital profit per admission, mean ± SD				0.194
Yen	1,132,765 ± 242,120	1,323,064 ± 279,670	1,448,522 ± 813,897	
US$^a^	8,091 ± 1,729	9,450 ± 1,998	10,347 ± 5,814	
Hospital operating margin, median (IQR)	0.80 (0.72–0.82)	0.86 (0.81–0.90)	0.88 (0.82–0.90)	< 0.001
Hospital stay, median (IQR), day	4 (4–5)	5 (4–7)	7 (6–11)	< 0.001
	**Mono-segmentectomy**			
Hospital revenue per admission, mean ± SD				0.376
Yen	2,046,747 ± 158,485	2,136,441 ± 259,557	1,935,973 ± 463,720	
US$^a^	14,620 ± 1,132	15,260 ± 1,854	13,828 ± 3,312	
Hospital costs per admission, mean ± SD				< 0.001
Yen	360,556 ± 73,950	323,449 ± 172,763	224,745 ± 107,078	
US$^a^	2,575 ± 528	2,310 ± 1,234	1,605 ± 765	
Hospital profit per admission, mean ± SD				0.696
Yen	1,686,191 ± 172,074	1,812,992 ± 252,383	1,711,228 ± 396,504	
US$^a^	12,044 ± 1,229	12,950 ± 1,803	12,223 ± 2,832	
Hospital operating margin, median (IQR)	0.85 (0.81–0.86)	0.89 (0.89–0.89)	0.90 (0.88–0.91)	< 0.001
Hospital stay, median (IQR), day	4 (3–4)	4 (4–6)	13 (8–13)	< 0.001
	**Resection of two contiguous sections**			
Hospital revenue per admission, mean ± SD				0.043
Yen	2,482,278 ± 304,697	NA	2,184,874 ± 333,695	
US$^a^	17,731 ± 2,176	NA	15,606 ± 2,384	
Hospital costs per admission, mean ± SD				0.007
Yen	619,055 ± 543,836	NA	244,003 ± 99,838	
US$^a^	4,422 ± 3,885	NA	1,743 ± 713	
Hospital profit per admission, mean ± SD				0.166
Yen	1,863,224 ± 824,141	NA	1,940,871 ± 284,440	
US$^a^	13,309 ± 5,887	NA	13,863 ± 2,032	
Hospital operating margin, median (IQR)	0.85 (0.79–0.85)	NA	0.89 (0.87-0.90)	0.006
Hospital stay, median (IQR), day	4 (4–4)	NA	9 (9–13)	< 0.001

Abbreviations: RLR, robotic liver resection; LLR, laparoscopic liver resection; OLR, open liver resection; SD, standard deviation; IQR, interquartile range; NA, not applicable

^a^ Calculated on an exchange rate of 140 yen to the dollar (the average from 2022 to 2024)

The economic evaluation, including the estimated intraoperative surgeon labor costs and estimated depreciation costs per surgery, is shown in Table S3 (Online Resource 5). The decrease in hospital profit was greater in the RLR group than in the LLR and OLR groups. This further decreased the hospital operating margin in the RLR group. The mean ± SD hospital profit per day was significantly different among the groups and significantly decreased from the RLR to LLR to OLR groups: RLR vs. LLR vs. OLR; single partial resection, 1,245 ± 406 US$ vs. 1,689 ± 627 US$ vs. 1,135 ± 583 US$ (*P* < 0.001 and P for trend < 0.001) (Fig. S2b [Online Resource 4]); mono-segmentectomy, 2,627 ± 546 US$ vs. 2,040 ± 691 US$ vs. 1,152 ± 429 US$ (*P* < 0.001 and P for trend < 0.001) (Fig. S2c [Online Resource 4]); RLR vs. OLR; resection of two contiguous sections, 2,422 ± 1275 US$ vs. 1,230 ± 328 US$ (*P* = 0.071) (Fig. S2d [Online Resource 4]).

## Discussion

This study is the first to evaluate the economic outcomes of RLR under the Japanese national health insurance system. The hospital revenue and profit per admission did not differ significantly among the three groups. However, hospital costs per admission were higher in the RLR group. The hospital profit per day was the highest in patients undergoing RLR, intermediate in those undergoing LLR, and the lowest in patients undergoing OLR, except for the subgroup including patients undergoing single partial resection. The findings of our study suggest that the higher total costs for RLR compared to those for LLR and OLR could be compensated by reduced additional healthcare costs and daily-cost efficiency owing to the lower complication rate and shorter length of hospital stay in patients undergoing RLR.

The reported medical costs associated with different liver resection approaches (i.e., RLR, LLR, and OLR) have shown considerable variability across studies. Some studies have found no significant difference in the total medical costs among the approaches [17, 18]. In contrast, several studies have shown that RLR is more expensive [6, 8, 10, 11]. Conversely, other studies have demonstrated that RLR is associated with lower medical costs [1, 7, 12, 15, 20]. These inconsistencies may reflect differences in the national healthcare systems, institutional cost structures, inclusion or exclusion of capital expenditures, patient selection criteria, and liver resection complexity. Therefore, cross-country comparisons of healthcare costs remain inherently challenging. Variations in the reimbursement systems, hospital accounting practices, labor costs, and the pricing of surgical equipment can significantly influence reported expenditures. Moreover, differences in healthcare infrastructures and resource allocation further complicate direct cost comparisons across international settings. In this context, we evaluated the economic outcomes in patients undergoing RLR, LLR, and OLR under the Japanese national health insurance system.

RLR has been regarded as increasing the surgical costs and potentially hindering hospital financial performance, particularly because of the high initial capital investment and maintenance costs associated with robotic platforms. However, our study showed that the financial impact of RLR is not necessarily negative. In fact, RLR may enhance the financial performance, particularly for complex liver resections. The mean hospital profit per day for patients undergoing RLR was comparable to that for patients undergoing LLR in single partial resection and notably more favorable in complex liver resections when compared to LLR and OLR (Fig. 1). This suggests that, despite higher initial capital investment, RLR may offer economic advantages in selected patient populations, procedural contexts, and well-occupied ward bed status. Under the Japanese DPC lump-sum payment system, the hospital revenue per admission is largely influenced by healthcare policy. In contrast, the hospital profit per day can be improved through surgeon-driven efforts, such as optimizing surgical procedures and postoperative management, thus potentially improving the hospital financial outcomes by increasing the surgical case volume and maintaining ward bed occupancy. The advantage of better outcomes and shorter length of hospital stay may not be ignored, especially when considering the hospital financial outcomes based on a ward basis, not a per patient basis.

Estimated intraoperative surgeon labor costs differed significantly among the groups, with the lowest costs in the RLR group. This reflects the reduced number of assistant surgeons in robotic surgery compared to laparoscopic and open surgery, offsetting longer operative times. In our group, it is standard practice to have two assistants (typically two fellows) or sometimes three assistants (typically adding one junior or senior resident) during OLR. This staffing level is necessitated by our institution’s dual role as a provider and educator. This setup facilitates maintaining the surgical field and performing suction from two different directions. We acknowledge that this may introduce some bias in estimating the surgeons’ labor costs at institutions where OLR is performed with fewer assistants. However, this bias is likely minimal because the mean estimated surgeon labor cost for the third assistant is 81 US$ (11,322 Japan Yen). Incorporating this adjustment decreases the mean estimated intraoperative surgeon labor cost for OLR from 648 US$ to 567 US$ (Table S3 [Online Resource 5]), but the overall findings of our study remain unchanged.

This study is associated with the following limitations. First, because our group did not use CUSA, the surgical costs of approximately US $ 700 (100,000 JPY) increased in institutions that used CUSA for liver resection. Second, we were unable to calculate the instrument costs for LLR or OLR procedures and the depreciation costs of laparoscopic systems. Nonetheless, we provided supplementary data of economic evaluations in the Online Resources, including the estimated intraoperative surgeon labor costs and estimated depreciation costs of robotic systems, to provide a more realistic evaluation of economic outcomes. Third, the impact of hospital profit per day may be influenced by the bed occupancy rate and sufficient patient volume. Fourth, the proportion of RLR among all liver resections increased annually from 2022 to 2024 (6% in 2022, 16% in 2023, and 27% in 2024); and thus, historical bias cannot be fully excluded for the assessment. Nonetheless, we have implemented our Enhanced Recovery After Surgery program, FASTOL program, since 2022 at our institution. Therefore, this bias may be minimal because the postoperative management, discharge criteria, and length of hospital stay were identical for both RLR and LLR cases. Last, this was a retrospective, single-institutional analysis conducted in a setting including initial RLR cases. As the OLR group included more technically complex procedures, such as resections for large tumors and cases involving vascular invasion, some selection bias cannot be completely eliminated. To ensure comparability between the groups, we excluded patients who underwent liver resection for perihilar cholangiocarcinoma, resections of two or more contiguous segments with vascular reconstruction, and resections of three contiguous segments (Fig. S1, Online Resource 2) because these procedures are not covered by the national insurance system for MILR or are rarely performed for LLR and RLR approaches. In addition, the application of IPTW substantially mitigated this bias, resulting in well-balanced baseline characteristics between groups. The significance of this study lies in the fact that it conducted an economic evaluation of RLR, which is currently in its early phase and is expected to become increasingly widespread in the coming years. At least in selected patient populations and under specific surgical procedures, it was shown that the financial impact of RLR is favorable.

In conclusion, the hospital revenue and profit per admission did not differ significantly among the three groups, although surgical charges were higher in patients undergoing MILR than in those undergoing OLR. RLR showed comparable hospital profit per day in single partial resection and superior performance in complex liver resection procedures when compared to LLR and OLR under the Japanese national health insurance system, suggesting potential economic advantages of RLR in selected patient populations, procedural contexts, and well-occupied ward bed status despite higher initial costs.

## Supplementary Information

Below is the link to the electronic supplementary material.

Supplementary Material 1

Supplementary Material 2

Supplementary Material 2

Supplementary Material 2

Supplementary Material 2

## Declarations

### Registry and the Registration No. of the study/trial

NA.
