# Correction: Daily cost-efficiency of robotic liver resection over laparoscopic and open liver resection especially for complex liver resection procedures: An inverse probability of treatment weighting analysis

**DOI:** 10.1007/s00595-026-03356-3

**Published:** 2026-06-16

**Authors:** Takeshi Kano, Yoshikuni Kawaguchi, Yujiro Nishioka, Akihiko Ichida, Takeshi Takamoto, Nobuhisa Akamatsu, Kiyoshi Hasegawa

**Affiliations:** Hepato-Biliary-Pancreatic Surgery Division, Department of Surgery, Graduate School of Medicine, The University of Tokyo, 7-3-1 Hongo, Bunkyo-ku, Tokyo, 113-8655 Japan

**Correction to: Surgery Today (2026) Published online April 22, 2026**

10.1007/s00595-026-03293-1

In the original publication the following things are missed out.

1. In PubMed, during the publication process, “Takeshi Kano” and “Kiyoshi Hasegawa” were incorrectly indicated as having contributed equally. The correct equal contributors are “Takeshi Kano” and “Yoshikuni Kawaguchi”.
2. In the PDF versions of Tables 1 and 2, the indentation for the subtype “Resection of two contiguous sections without vascular reconstruction” under “Disease type” was inadvertently removed during the publication process.
3. In the PDF versions of Tables 1 and 2, “Largest tumor diameter” and “Number of resection sites > 2” were incorrectly indented during the publication process, making them appear as subgroups under “Disease type”.
4. In Tables 1 and 2, from the original manuscript stage, the abbreviations “PHCC, perihilar cholangiocarcinoma” and “GBC, gallbladder cancer” were incorrectly included in the abbreviations for Disease type despite not being used in the tables. In addition, the order of abbreviations under Disease type was incorrect. The correct abbreviations are: “HCC, hepatocellular carcinoma; ICC, intrahepatic cholangiocarcinoma; CRLM, colorectal liver metastases; NET, neuroendocrine tumor”.
5. In the PDF version of Table 1, during the publication process, the space between “b” and “Excluding left lateral segmentectomy” in the abbreviations section was inadvertently omitted.
6. In Table 2, during the publication process, the first row containing “Characteristics,” “RLR group,” “LLR group,” “OLR group,” “SMD,” and “P value” was omitted.
7. In the PDF version of Table 3, during the publication process, the horizontal line separating the “Without adjustment” analysis and the “With adjustment” analysis was omitted, hindering the readability of the table.
8. In the PDF version of Table 3, during the publication process, “Postoperative hospital stay” and “30-day readmission” were incorrectly indented, making them appear as subgroups under “Morbidity Clavien–Dindo classification”.
9. In Table 5, during the publication process, the subgroup title “Single partial resection” was inadvertently inserted into the first-row heading “Characteristics”. In addition, in the PDF version, the horizontal lines between the subgroups “Single partial resection”, “Mono-segmentectomy”, and “Resection of two contiguous sections” were omitted, hindering the readability of the table.
10. In the legend of Fig. 1, from the original manuscript stage, the abbreviation ‘SD, standard deviation’ was incorrectly included.
11. In the Supplementary Information section, the links for Supplementary Materials 3, 4, and 5 were incorrectly labeled as ‘Supplementary Material 2’ during the publication process. In addition, figures and tables with revisions highlighted in red, which had been submitted during the revision process, were used in the published version.

The original article has been corrected.

