# Supplementary material for: Daily cost-efficiency of robotic liver resection over laparoscopic and open liver resection especially for complex liver resection procedures: An inverse probability of treatment weighting analysis
Source: Surg Today. 2026 Apr 22;56(9):1868–78. doi: 10.1007/s00595-026-03293-1 (PMC13473331; doi:10.1007/s00595-026-03293-1)
Supplement: Supplementary file 1 [file 595_2026_3293_MOESM1_ESM.pdf]

Table S1. Surgical charges of open and minimally invasive liver resection.

No.	Liver resection procedures	Surgical charges (US$[a])	
		OLR	MILR
1	Partial liver resection		
a	Single resection	3,096	4,549
b	Multiple partial resection	3,295	5,349
2	Mono–segmentectomy	4,502	7,773
3	Left lateral sectionectomy	2,717	4,191
4	Resection of one section[b] (excluding left lateral sectionectomy)	4,336	9,338
5	Resection of two contiguous sections[c] without vascular reconstruction	5,444	10,889
6	Resection of three contiguous sections or more[d]	6,931	12,435
7	Resection of two contiguous sections[c] or more with vascular reconstruction	9,016	Not available[e]

Abbreviations: OLR, open liver resection; MILR, minimally invasive liver resection.

[a] Calculated on an exchange rate of 140 yen to the dollar (the average from 2022 to 2024)

[b] Resection of one section (excluding left lateral sectionectomy) includes: left median sectionectomy (segment 4), right median sectionectomy (segments 5 and 8) and right posterior sectionectomy (segments 6 and 7)

[c] Resection of two contiguous sections includes: left hepatectomy (segments 2, 3, and 4 with or without segment 1), right hepatectomy (segments 5, 6, 7, and 8 with or without segment 1), central hepatectomy (segments 4, 5, and 8)

[d] Resection of three contiguous sections or more includes: extended right hepatectomy (segments 4, 5, 6, 7, and 8 with or without segment 1), extended left hepatectomy (segments 2, 3, 4, 5, and 8 with or without segment 1)

[e] Resection of two contiguous sections or more with vascular reconstruction is not covered by the Japanese national health insurance system as of April 2025 for laparoscopic and robotic liver resections.
