# Supplementary material for: Daily cost-efficiency of robotic liver resection over laparoscopic and open liver resection especially for complex liver resection procedures: An inverse probability of treatment weighting analysis
Source: Surg Today. 2026 Apr 22;56(9):1868–78. doi: 10.1007/s00595-026-03293-1 (PMC13473331; doi:10.1007/s00595-026-03293-1)
Supplement: Supplementary file 2 [file 595_2026_3293_MOESM2_ESM.pdf]

Fig. S1 Study flow chart 402 patients who underwent liver resection from 2022 to 2024

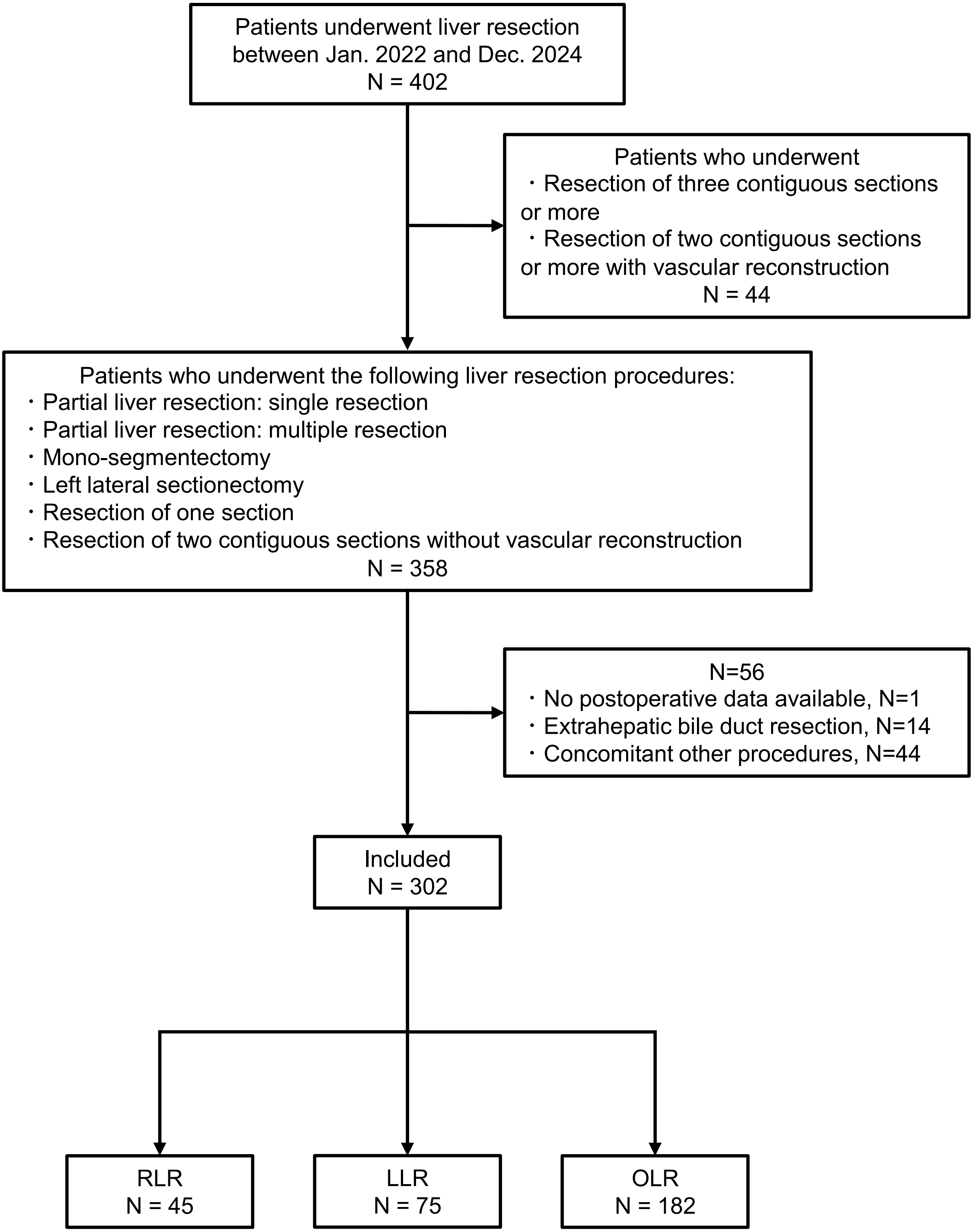

Abbreviations: RLR, robot liver resection; LLR, laparoscopic liver resection; OLR, open liver resection.
