# Supplementary material for: Daily cost-efficiency of robotic liver resection over laparoscopic and open liver resection especially for complex liver resection procedures: An inverse probability of treatment weighting analysis
Source: Surg Today. 2026 Apr 22;56(9):1868–78. doi: 10.1007/s00595-026-03293-1 (PMC13473331; doi:10.1007/s00595-026-03293-1)
Supplement: Supplementary file 3 [file 595_2026_3293_MOESM3_ESM.pdf]

Table S2. Overall economic outcomes by liver resection approach including intraoperative surgeon labor costs and depreciation expenses of robotic systems after inverse-probability of treatment weighted adjustment.

Characteristics	RLR group	LLR group	OLR group	P value
Intraoperative surgeon labor costs, mean ± SD				< 0.001
Yen	70,091 ± 20,570	59,836 ± 27,439	90,656 ± 40,827	
US$[a]	501 ± 158	427 ± 196	648 ± 292	
Hospital profit, mean ± SD				< 0.001
Yen	1,171,947 ± 433,452	1,413,824 ± 485,150	1,519,413 ± 547,719	
US$[a]	8,371 ± 3,096	10,099 ± 3,465	10,853 ± 3,912	
Hospital operating margin, median (IQR)	0.61 (0.58–0.62)	0.81 (0.77–0.85)	0.83 (0.80–0.85)	< 0.001

Abbreviations: RLR, robotic liver resection; LLR, laparoscopic liver resection; OLR, open liver resection; SD, standard deviation; IQR, interquartile range

[a] Calculated on an exchange rate of 140 yen to the dollar (the average from 2022 to 2024)
