# Supplementary material for: Daily cost-efficiency of robotic liver resection over laparoscopic and open liver resection especially for complex liver resection procedures: An inverse probability of treatment weighting analysis
Source: Surg Today. 2026 Apr 22;56(9):1868–78. doi: 10.1007/s00595-026-03293-1 (PMC13473331; doi:10.1007/s00595-026-03293-1)
Supplement: Supplementary file 4 [file 595_2026_3293_MOESM4_ESM.pdf]

Fig. S2 Hospital profit per day including estimated intraoperative surgeon labor costs and estimated depreciation costs of robotic platform after inverse-probability of treatment weighted adjustment

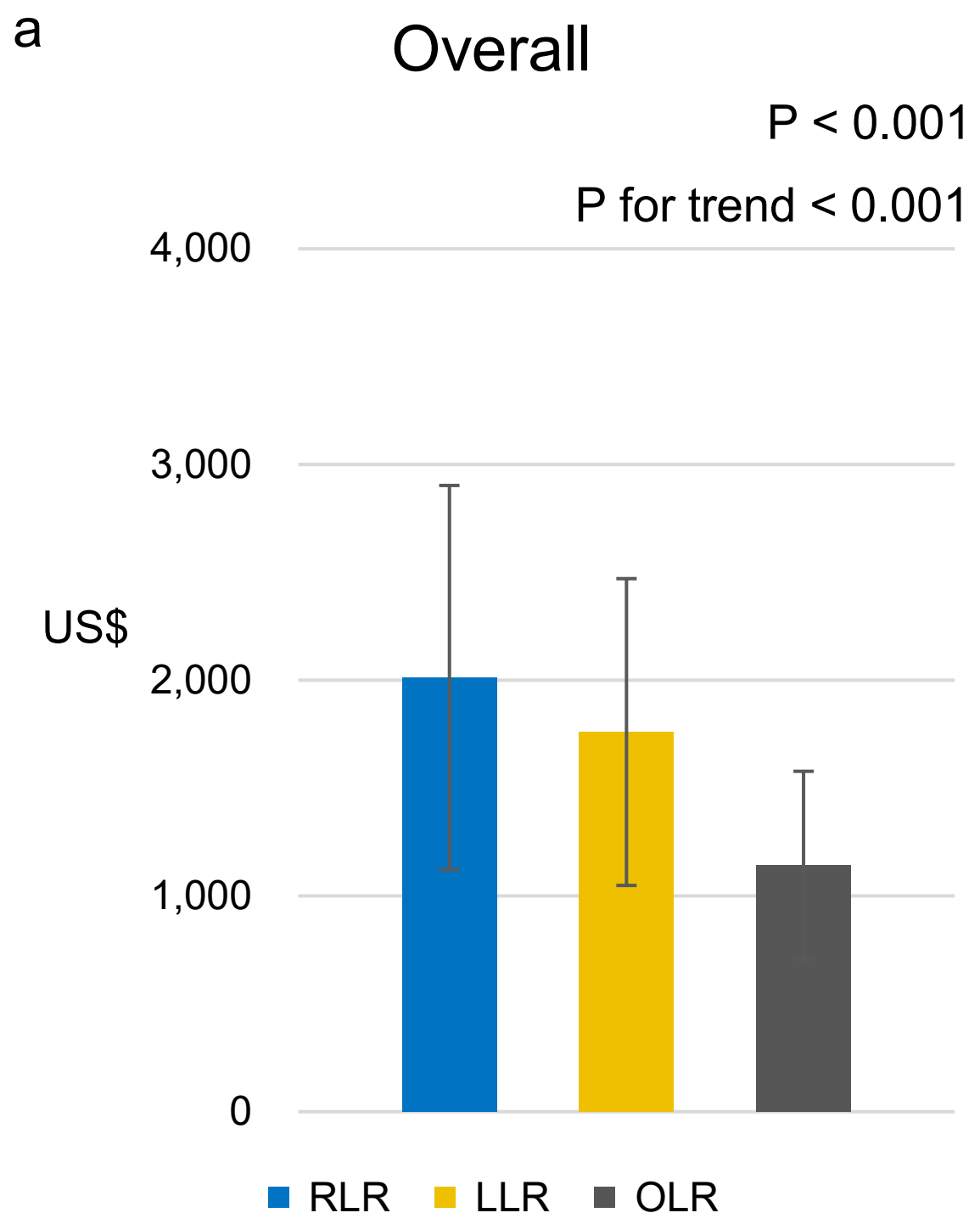

(a) RLR vs. LLR vs. OLR; Overall cohort, 2,013 ± 890 US$ vs. 1,760 ± 711 US$ vs. 1,142 ± 436 US$ (P < 0.001 and P for trend < 0.001)

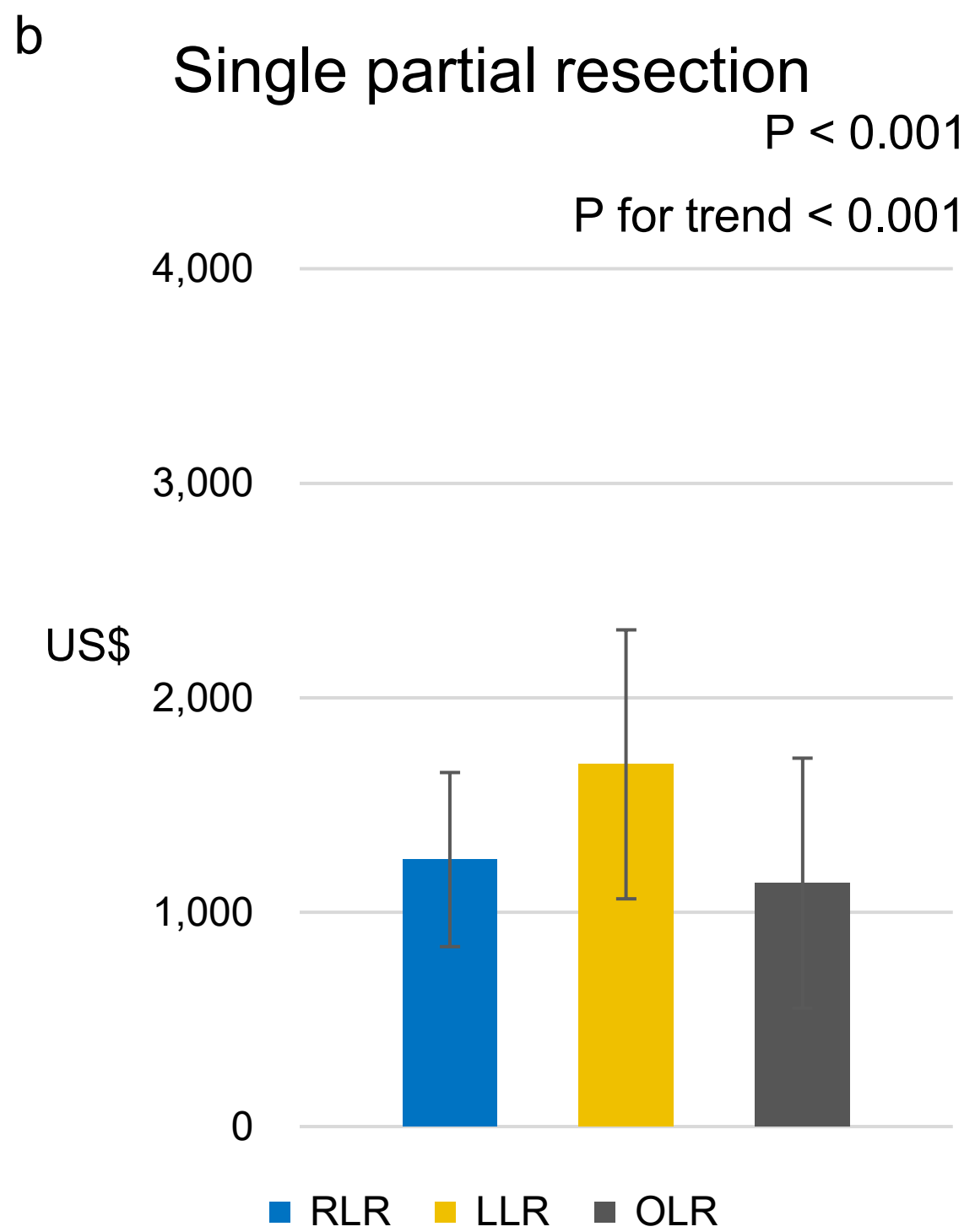

(b) RLR vs. LLR vs. OLR; Single partial resection, 1,245 ± 406 US$ vs. 1,689 ± 627 US$ vs. 1,135 ± 583 US$ (P < 0.001 and P for trend < 0.001)

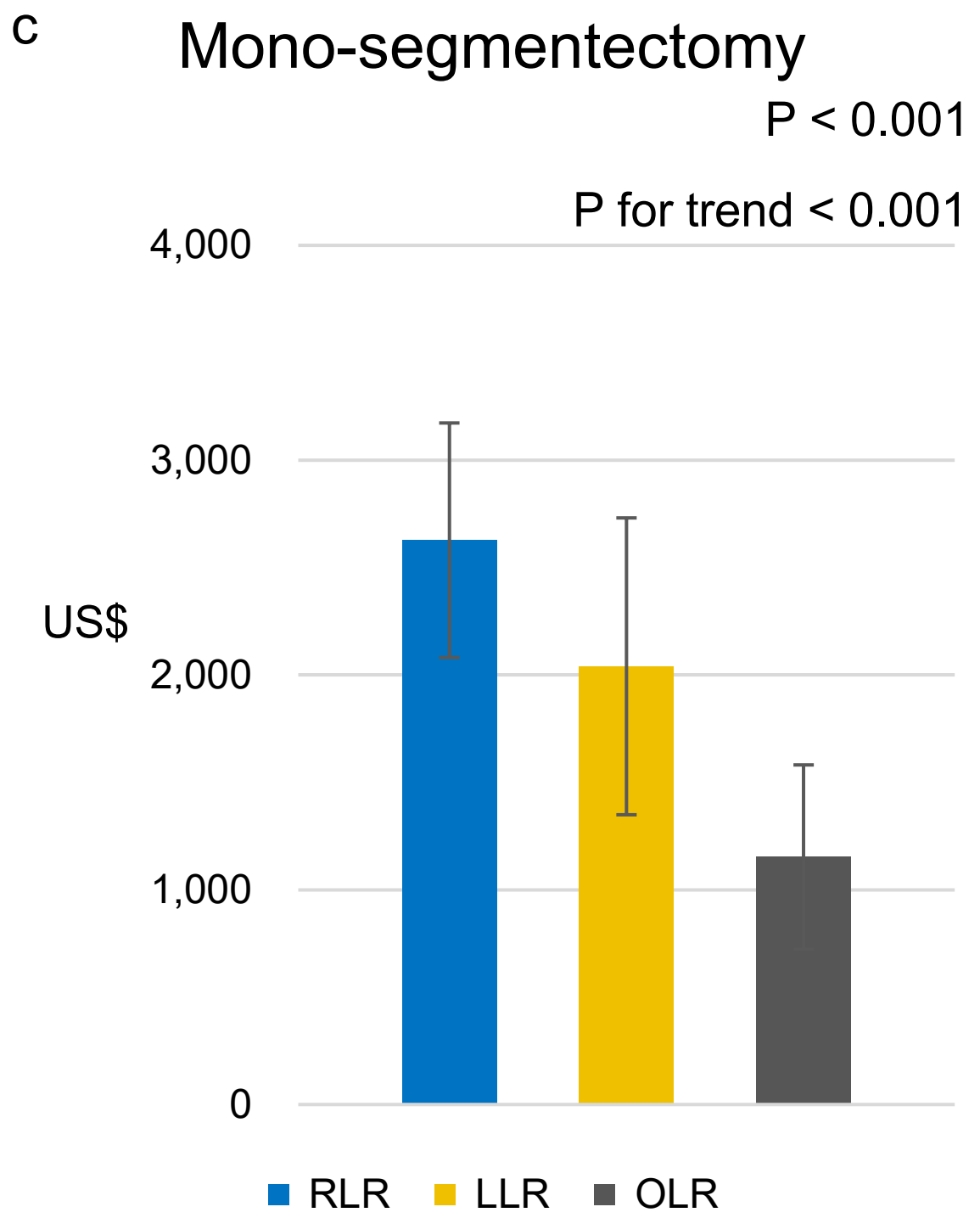

(c) RLR vs. LLR vs. OLR; Mono-segmentectomy, 2,627 ± 546 US$ vs. 2,040 ± 691 US$ vs. 1,152 ± 429 US$ (P < 0.001 and P for trend < 0.001)

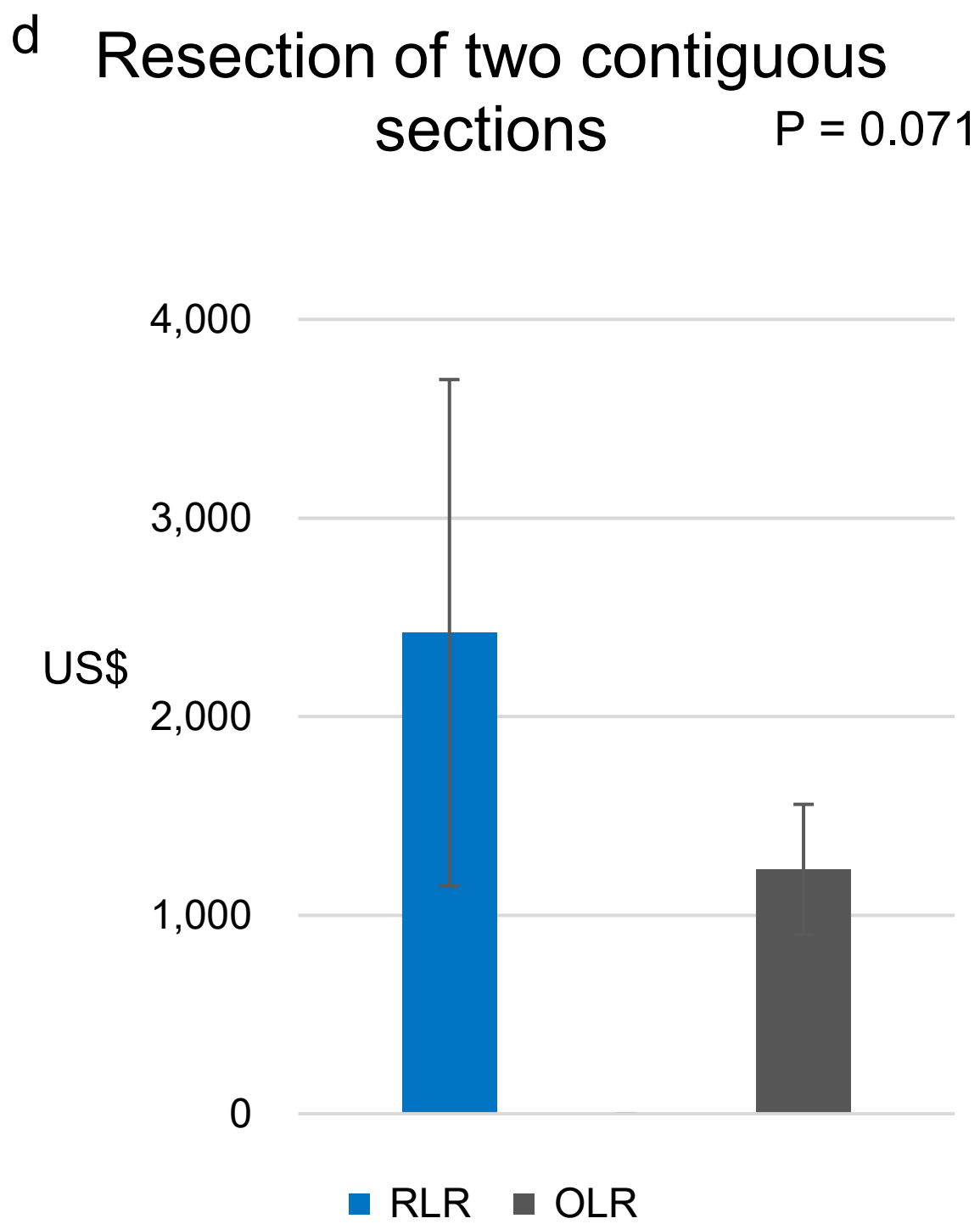

(d) RLR vs. OLR; resection of two contiguous sections, 2,422 ± 1275 US$ vs. 1,230 ± 328 US$ (P =0.071)

Abbreviations: RLR, robot liver resection; LLR, laparoscopic liver resection; OLR, open liver resection; SD, standard deviations.
