# Supplementary material for: Daily cost-efficiency of robotic liver resection over laparoscopic and open liver resection especially for complex liver resection procedures: An inverse probability of treatment weighting analysis
Source: Surg Today. 2026 Apr 22;56(9):1868–78. doi: 10.1007/s00595-026-03293-1 (PMC13473331; doi:10.1007/s00595-026-03293-1)
Supplement: Supplementary file 5 [file 595_2026_3293_MOESM5_ESM.pdf]

Table S3. Economic outcomes by liver resection procedure types including intraoperative surgeon labor costs and depreciation expenses of robotic systems after inverse-probability of treatment weighted adjustment.

Characteristics	RLR group	LLR group	OLR group	P value
		**Single partial resection**		
Intraoperative surgeon labor costs, mean ± SD				0.023
Yen	61,047 ± 19,989	50,360 ± 22,341	68,940 ± 43,272	
US$[a]	436 ± 143	360 ± 160	492 ± 309	
Hospital profit, mean ± SD				< 0.001
Yen	838,399 ± 249,986	1,272,704 ± 274,508	1,379,583 ± 795,420	
US$[a]	5,989 ± 1,786	9,091 ± 1,961	9,854 ± 5,682	
Hospital operating margin, median (IQR)	0.61 (0.59–0.62)	0.83 (0.78–0.86)	0.83 (0.78–0.85)	< 0.001
		**Mono-segmentectomy**		
Intraoperative surgeon labor costs, mean ± SD				0.177
Yen	70,431 ± 17,995	85,179 ± 33,599	90,374 ± 38,597	
US$[a]	503 ± 129	608 ± 240	646 ± 276	
Hospital profit, mean ± SD				0.062
Yen	1,392,425 ± 156,416	1,694,482 ± 320,106	1,620,853 ± 393,893	
US$[a]	9,946 ± 1,117	12,103 ± 2,286	11,578 ± 2,814	
Hospital operating margin, median (IQR)	0.70 (0.66–0.71)	0.85 (0.85–0.85)	0.87 (0.83–0.87)	< 0.001
		**Resection of two contiguous sections**		
Intraoperative surgeon labor costs, mean ± SD				0.685
Yen	92,031 ± 23,137	NA	102,452 ± 34,029	
US$[a]	657 ± 165	NA	732 ± 243	
Hospital profit, mean ± SD				0.041
Yen	1,537,874 ± 803,224	NA	1,838,419 ± 272,691	
US$[a]	10,985 ± 5,737	NA	13,132 ± 1,948	
Hospital operating margin, median (IQR)	0.73 (0.65-0.73)	NA	0.83 (0.82-0.86)	< 0.001

Abbreviations: RLR, robotic liver resection; LLR, laparoscopic liver resection; OLR, open liver resection; SD, standard deviation; IQR, interquartile range; NA, not applicable

[a] Calculated on an exchange rate of 140 yen to the dollar (the average from 2022 to 2024)
